# Identifying the start of a platelet aggregate by the shear rate and the cell-depleted layer

**DOI:** 10.1098/rsif.2019.0148

**Published:** 2019-10-02

**Authors:** B. J. M. van Rooij, G. Závodszky, V. W. Azizi Tarksalooyeh, A. G. Hoekstra

**Affiliations:** Computational Science, Institute for Informatics, University of Amsterdam, Amsterdam, The Netherlands

**Keywords:** thrombosis, cell-based model, vasoconstriction, platelet margination, lattice Boltzmann method

## Abstract

Computer simulations were performed to study the transport of red blood cells and platelets in high shear flows, mimicking earlier published *in vitro* experiments in microfluidic devices with high affinity for platelet aggregate formation. The goal is to understand and predict where thrombus formation starts. Additionally, the need of cell-based modelling in these microfluidic devices is demonstrated by comparing our results with macroscopic models, wherein blood is modelled as a continuous fluid. Hemocell, a cell-based blood flow simulation framework is used to investigate the transport physics in the microfluidic devices. The simulations show an enlarged cell-depleted layer at the site where a platelet aggregate forms in the experiments. In this enlarged cell-depleted layer, the probability to find a platelet is higher than in the rest of the microfluidic device. In addition, the shear rates are sufficiently high to allow for the von Willebrand factor to elongate in this region. We hypothesize that the enlarged cell-depleted layer combined with a sufficiently large platelet flux and sufficiently high shear rates result in an haemodynamic environment that is a preferred location for initial platelet aggregation.

## 1. Introduction

Arterial thrombosis occurs in vessels with a pathological shear rate (greater than 5000 s^−1^) caused by for example a stenosed vessel due to atherosclerosis [1] or by disturbed flow around medical device [2]. Two other factors that are important for arterial thrombosis are a prothrombotic surface and prothrombotic blood chemistry (e.g. specific behaviour of von Willebrand factor (vWF) and platelets) [3]. In comparison with venous thrombosis, the typical coagulation cascade is less important in arterial thrombosis. The reason for this is that the flow is faster in arteries and the coagulation cascade happens on a longer timescale (minutes) than the aggregation of platelets (seconds) in these fast flows. Different types of clots can be distinguished in the venous and arterial system. Red blood cell-rich clots or red clots are mostly found in the veins and platelet-rich clot or white clots are found in the arteries. An increase in local shear rate can result in shear-induced platelet aggregation [2] which can lead to occlusion of the vessel or medical device. At high shear rates the vWF uncoils [4]. This uncoiling can happen when the vWF is free flowing in the blood or when it is bound to a prothrombotic surface (collagen). The shear rate threshold for the uncoiling of free-flowing vWF is around 5000 s^−1^ [4] and for bounded vWF it is around 1000 s^−1^ [5]. When the vWF is uncoiled, binding sites (A1 domain) become available for platelet receptors (GP Ib-V-IX or integrins), collagen and endothelial cells [6]. The vWF slows the platelets down to facilitate the binding of a platelet to collagen or platelet–integrin binding to vWF, forming a stable platelet aggregate [7].

In the last decade, many *in vivo* and *in vitro* experiments on the formation of a platelet aggregate in a vessel or microfluidic device with a stenosis were performed, e.g. [8–10] Nesbitt *et al*. [8] show that rapid changes in shear rate caused by stenosis or by the developing thrombus itself lead to the formation of a platelet aggregate. Westein *et al*. [10] extend this explanation by accounting for the importance of the vWF during platelet aggregation at post-stenosis sites. The influence of red blood cells (RBCs) on the formation of a platelet aggregate was investigated by Tovar-Lopez *et al*. [11] by dividing the flow in two fractions with different haematocrit. This study showed that the platelets present in the platelet aggregate came from 15 to 25% of the blood flow proximal to the stenosis. This addresses the importance of platelet margination in thrombus formation.

The shear rate and its gradients in the microfluidic devices used in those experiments were simulated by a model that represents blood as a continuous fluid. Many papers on experimental thrombosis have explored the haemodynamical parameters by computational fluid dynamics using continuous models [8–10,12,13]. A primary drawback of this method is that the influence of RBC is ignored. Given the fact that the size of the initial platelet aggregates is smaller than individual RBC, and that the scale of the microfluidic devices are typically only one order of magnitude larger than a red blood cell a full interpretation of the flow fields in these experimental set-ups would require computations that explicitly take the RBC and platelets into account. Such computations reproduce influential phenomena of blood, such as cell-depleted layers and platelet margination. The impact of RBC on the transport physics at the site of platelet aggregate initialization is not clearly understood yet. Tovar-Lopez *et al*. [11] hypothesize that collisions between RBCs and platelets are a key mechanism in the formation of the platelet aggregate in the lower shear zone behind the stenosis, but they did not further explore this hypothesis.

In this study, a cell-resolved model [14] that includes the behaviour of RBC and platelets is used. Many cell-resolved models have been developed in the last decade. The mostly used approaches for these models that can be found in the literature are the lattice Boltzmann method (LBM) in combination with the immersed boundary method (IBM) [14–17] and the dissipative particle method [18–25]. Additionally, the most popular material model for red blood cell deformation is the model of Fedosov *et al*. [19]. However, in this study, the model developed by Závodszky *et al*. [14] is used, since this model performs better at high shear rates and the transport physics in microfluidic devices that display very high shear rates (1000–40 000 s^−1^) is investigated. The computational costs of the cell-resolved models are extremely high and therefore only small domains and short time spans can be studied with these models. Cell-based models can be used to study the suspension behaviour of blood with phenomena, such as the cell-depleted layer [26,27] and the margination of platelets [24,27,28]. The increased concentration of platelets at the side of the vessel wall is an important factor in arterial thrombosis. Additionally, these models can be of great value by developing experiments or optimizing microscale medical devices.

A direction of thrombosis research that recently gained more interest is the dynamics of the vWF that play a large role in high shear thrombosis. Rack *et al*. [29] and Lui *et al*. [30] included the vWF in their cell-based model to study the behaviour of this protein in the blood. Griffin *et al*. try to find new anti-thrombotic drugs in the form of nanoparticles that reduce the amount of uncoiling of vWF at high shear rates [31]. Additionally, Belyaev modelled the binding of platelets and vWF [32]. Furthermore, experimentalists try to understand the behaviour of the vWF better by designing experiments that specifically target high shear rate regimes [5,12]. Despite all the recent developments, the influence of RBC on the uncoiling of the vWF and in what haemodynamic environment this uncoiling happens is still unknown.

In this study, the transport of platelets in complex microfluidic geometries is investigated using computer simulations to shed further light on the role of haemodynamics and transport physics in initial platelet aggregation. For this, we rely on our cell-based blood flow simulation framework Hemocell [14]. The mechanical behaviour and transport properties of the RBC in Hemocell were thoroughly validated in Zadvodszky *et al*. [14]. First, the cell-resolved flow dynamics of whole blood in flow chambers with stenosis is studied, i.e. the experiments of Tovar-Lopez *et al*. [9] are reproduced. Next, the thickness of the cell-depleted layers is investigated in detail and the platelet residence time as well as the platelet density in these layers are measured. The fluid shear rate and shear stresses are studied as well. Combining all this information, the aim is to better understand and predict where platelet aggregate formation starts. Additionally, the method used and results found in this study can be useful for designing future experiments and for optimizing the design of micro-medical devices.

## 2. Material and methods

### 2.1. Cell-based blood flow framework: Hemocell

Hemocell [14,33] is used to simulate the cell-resolved flow of blood in *in vitro* experiments in which a platelet aggregate is formed. Within Hemocell, the blood plasma is modelled as an incompressible fluid by the LBM using the Palabos library [34,35]. The suspended RBC and platelets are modelled by a discrete element method (DEM) and fluid–structure coupling is achieved via the IBM. The RBC membrane model by Zavodszky *et al*. [14] is used to mimic the deformations of the RBC in the flow. The constitutive model for cells is validated by the optical tweezers stretching test of Mills *et al*. [36] and the wheeler shear test of Yao *et al*. [37]. In addition, the rheology of blood is validated using cell-free layer (CFL) measurements [38], velocity profile measurements [39] and the Pries curve [40]. This model was found to be more suitable to model red blood cell mechanics in high shear environments [14]. For further details on the material model and its validations the reader is referred to [14].

### 2.2. Experiments from the literature

**Figure 1.**
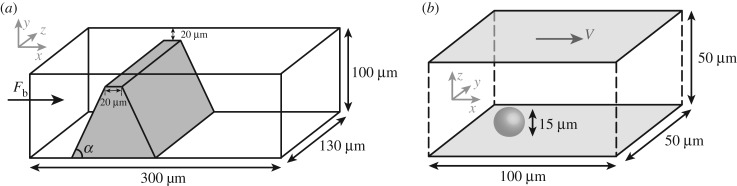
Geometry of the microfluidic devices: (*a*) microcontraction experiment of Tovar-Lopez *et al*. [9], in the figure the contraction angle of the microfluidic device is given by *α* (30, 60 or 90°) and the body force *F*_b_ drives the flow. (*b*) Bead assay experiment of Nesbitt *et al*. [8], the geometry of the largest bead (15 μm) is shown and in this simulation the flow is driven by a velocity (*V*) boundary at the top plane of the geometry.

In this paper, two experiments from the literature are examined in more detail using Hemocell. First, the microfluidic experiment of Tavar-Lopez *et al*. [9] is reproduced. The geometry of the experiment as used in our simulations is shown in figure 1*a*. In this experiment, the development of a platelet aggregate in time is studied using a microfluidic channel with a microcontraction. Different contraction/expansion angles *α* of 90, 60 and 30°, that obstruct 80% of the microfluidic device, are investigated. In the numerical reproduction of the experimental set-up, Tovar-Lopez *et al*. [9] applied a continuous Newtonian fluid model to the geometry and they reported shear rates at 1 μm from the wall. In this work, the blood flow in these geometries was studied using a continuous fluid model, to reproduce Tovar-Lopez’s results, and Hemocell with periodic boundary conditions in accordance with the corresponding flow profile. The top, bottom, front and back plane of the device are simulated as solid walls with no-slip boundary conditions.

The bead assay experiment performed by Nesbitt *et al*. [8] is the second experiment that is studied in this paper (figure 1*b*). A glass sphere (diameter = 2, 5, 9 or 15 μm) coated with vWF was attached to the bottom of a flow channel (0.2 × 2.0 mm) which is perfused with whole blood. Nesbitt *et al*. varied the size of the sphere to study the influence of the micro-gradient in shear rate on the aggregation size. A part of this flow chamber domain is simulated in Hemocell (50 × 50 × 100 μm) and periodic boundary conditions are used in the *x*- and *y*-directions (figure 1*b*). On the top of the domain, a velocity is prescribed to drive the flow. The sphere and bottom of the channel are modelled as no-slip walls.

### 2.3. Simulation parameters

For the microcontraction simulations, approximately the same input parameters as those used by Tovor-Lopez *et al*. [9] are used here. The volumetric flow rate of approximately 16 μl min^−1^ is obtained in our simulations by driving the flow with the corresponding body force (*F*_b_, figure 1*a*). The flow rate for the 60° case is 17 μl min^−1^. The discharge haematocrit is 36% and the kinematic blood viscosity in the simulation where blood is modelled as a continuous fluid is set to 3.3 m^2^ s^−1^. The simulations with blood modelled as a continuous fluid and blood modelled as a suspension are driven with the same flow rate. The contraction angles *α* (figure 1*a*) are chosen the same as in the experiment [9]: 30, 60 and 90°. In the cell-resolved simulations, the platelets and RBC are initially randomly distributed with a platelet to RBC number ratio of 1 : 10, and we allow for stable cell distribution during a period of 20 ms that is needed to reach a steady state in viscosity [14]. The random distribution of RBC and platelets is performed by a packing algorithm build in Hemocell [14]. Note that these simulations, simulating blood flow at high shear rates and haematocrit of 36% are at the upper limit of what is currently achievable with cell-based models like Hemocell [14]. Also, as these simulations typically contain O(10^4^) cells, we need to execute the simulations on parallel supercomputers. We typically ran the 60° microcontraction simulation with 8444 RBCs and 711 platelets on 256 cores of the supercomputer Cartesius (Amsterdam, The Netherlands) (SURFsara. Cartesius) for 5 days.

For the bead assay experiment, two simulations with different initial velocities were performed. Shear rates of 675 s^−1^ and 1875 s^−1^ were applied on the velocity boundary at the top plane of the domain. A discharge haematocrit of 25% and sphere diameters of 2 and 15 μm were used. The platelets are fully marginated at the start of the simulation. The bead assay computation with the large bead contained 598 RBCs and 68 platelets and was computed on 64 cores of Cartesius (SURFsara. Cartesius) for 10 days. The small bead assay computation contained 637 RBCs and 73 platelets and was computed on 64 cores for 10 days.

The parameters for the blood plasma and for the red blood cell model are the same for both experiments and these can be found in the electronic supplementary material.

### 2.4. Cell-free layer and haematocrit

The time-averaged CFL is obtained by measuring the local concentration of RBC in the domain averaged over all time steps. If the local concentration is lower than a certain threshold, this is defined as the CFL. The threshold used in this study is 10% of the volume fraction.

**Figure 2.**
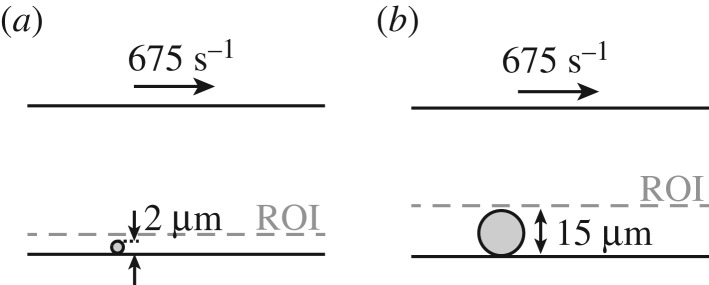
The region of interest is the area below the dashed line in which the platelet count per second, residence time of a platelet and shear rate are averaged in the bead assay experiment for the 2 μm bead (*a*) and the 15 μm bead (*b*).

The region of interest (ROI) in the bead assay simulations in which the platelet count per second, residence time of a platelet and shear rate are measured is the area from the bottom plate to 3 μm above this plate for the small bead (2 μm, figure 2*a*) and the area from the bottom plate to 16 μm above this plate for the large bead (15 μm, figure 2*b*).

The residence time of platelet *τ*_*r*_ is defined as the time it takes for a platelet to move a diameter away from the current position in space

2.1 $$\tau_{r}=\frac{D_{\mathrm{plt}}}{V_{\;f}},$$

with *D*_plt_ the diameter of platelet (2 μm) and *V*_*f*_ the time-averaged fluid velocity at the position of the platelet.

## 3. Results

In the following sections, the results of our study of the transport physics in devices that favour platelet aggregate formation are presented. We start with the microcontraction experiment in which we investigate the difference between continuous and cell-resolved modelling. Second, we study the haemodynamic environment in which the platelet aggregate starts to form in the bead assay experiment.

### 3.1. Continuous versus cell-resolved blood flow modelling

**Figure 3.**
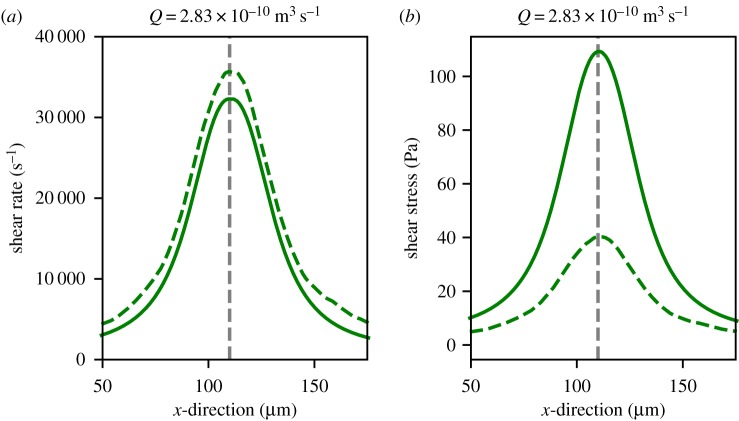
(*a*) Shear rate and (*b*) shear stress obtained 1 μm above the contraction wall in the 60° geometry. The dashed line presents the simulation with blood modelled as a suspension (Hemocell) and the solid line presents the simulation with blood modelled as a continuous fluid. The vertical grey dashed line is the position of the apex of the microcontraction in the microfluidic device. (Online version in colour.)

In this subsection, the shear rates and shear stresses of the microcontraction for blood modelled as a continuous fluid and blood modelled as a suspension are compared. In our simulations, the microcontraction is perfused either with a continuous fluid or a suspension of RBC and platelets in blood plasma. The resulting shear rates and stresses of the fluid (as defined in the electronic supplementary material) that a platelet or vWF will feel when it moves 1 mm from the wall of the microfluidic device are shown in figure 3*a*,*b*, respectively.

Figure 3*a* shows that for the 60° microcontraction simulation a 10% higher shear rate was found when the blood was simulated as a suspension (Ht =36%). The increase in shear rate was even more pronounced in the simulation with a haematocrit of 44% (data not shown). We approximately reproduced the shear rates as those reported by Tovar-Lopez *et al*. [9] in our continuous fluid simulations.

However, the shear stress is significantly higher in the continuous fluid simulation in comparison to the cell-based simulation. This can be explained by the fact that the shear stress was obtained in the CFL. In this layer, the local viscosity is thrice as low as in the continuous fluid simulation which leads to a higher shear stress in the continuous simulation.

### 3.2. An enlarged cell-depleted layer

**Figure 4.**
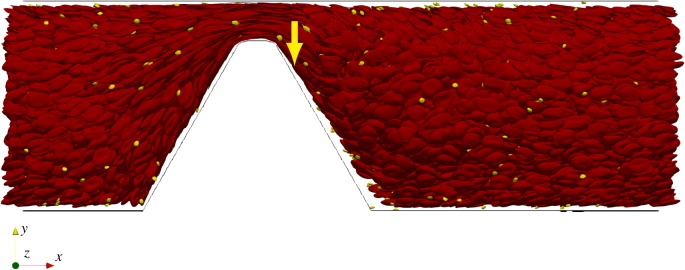
Side view of a snapshot of the 60° microcontraction simulation with blood modelled as a suspension. The red blood cells are coloured red and the platelets are coloured yellow. The yellow arrow indicates the position where the platelet aggregate started to form in the experiment of Tovar-Lopez *et al*. [9]. A cell-free layer is clearly visible on the top and bottom of the constricted flow channel. (Online version in colour.)

In our simulation of the experiment of Tavor-Lopez *et al*. [9], we observed an enlarged CFL at the location where the platelet aggregate started to form. A visualization of this simulation is presented in figure 4. The yellow arrow points to the position where the platelet aggregate approximately started to form as reported by Tovar-Lopez *et al*. [9]. At this place, the cell-depleted layer becomes larger. Such an enlarged CFL was observed for all three geometries used in the experiment of Tavor-Lopez (see electronic supplementary material).

**Figure 5.**
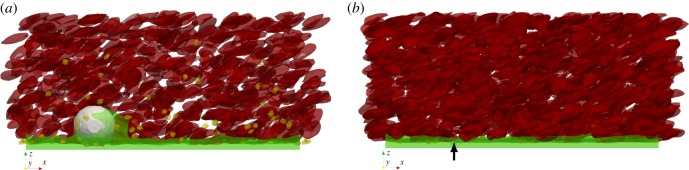
Side view of the bead assay experiment with beads of the size of (*a*) 15 μm and (*b*) 2 μm. In (*a*), a part of the cells are clipped from the domain to make the bead visible in the figure. The time-averaged cell-free layer is coloured green on the bottom of the flow channel. The red blood cells (red) and platelet (yellow) are taken from one time step and added to the image. The haematocrit in this simulation was 25% and flow is driven by a shear rate of 675 s^−1^ on the top plane. The black arrow is pointing to the small bead to better visualize it. (Online version in colour.)

The averaged cell-depleted layer was also larger in the bead assay simulation with the largest sphere (15 μm), which for a haematocrit of 25% is shown in green in figure 5*a*. The smaller sphere with a diameter of 2 μm is completely embedded in the CFL as shown in figure 5*b*.

### 3.3. Platelet count per second and platelet residence time

**Figure 6.**
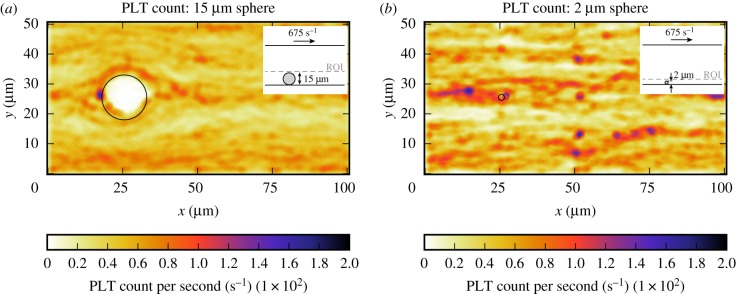
Two-dimensional projection of the averaged number of platelets that are present per second in the bottom layer of the flow chamber (ROI) of the bead assay simulation of the largest sphere (*a*) and smallest sphere (*b*) ($\dot{\gamma }=675\;{\text{s}}^{-1}$). The position of the beads are marked by the black circle. The simulation details are shown in the inset image. The dashed line gives the upper limit of the layers in which the platelet count per second is averaged. (Online version in colour.)

**Figure 7.**
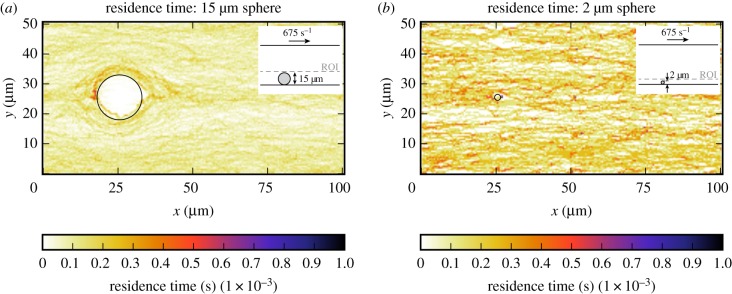
Two-dimensional projection of the residence time of platelets in the bottom layer of the flow chamber (ROI) of the bead assay simulation of the largest sphere (*a*) and smallest sphere (*b*) ($\dot{\gamma }=675\;{\text{s}}^{-1}$). The position of the sphere is marked by the black circle. The simulation details are shown in the inset image. The dashed line gives the upper limit of the layers in which the PLT residence time is averaged. (Online version in colour.)

**Figure 8.**
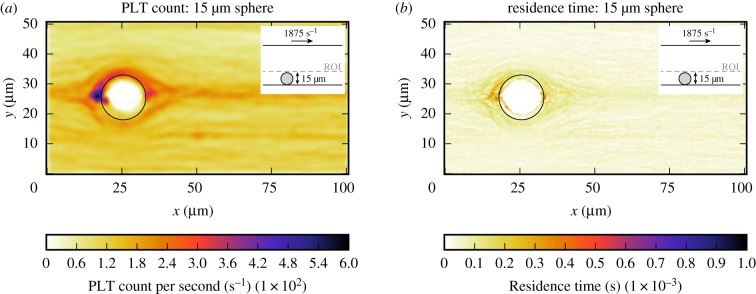
Two-dimensional projection of the count of platelets per second (*a*) and residence time of platelets (*b*) in the bottom layer of the flow chamber (ROI) of the bead assay simulation of the largest sphere ($\dot{\gamma }=1875\;{\text{s}}^{-1}$). The position of the sphere is marked by the black circle. The simulation details are shown in the inset image. The dashed line gives the upper limit of the layers in which the parameters are averaged. (Online version in colour.)

In this section, the platelet count per second and residence time of a platelet in the CFLs of the bead assay experiment are obtained. Figures 6 and 7 show the projection to two dimensions of the platelet count per second and the projection of the residence time of platelets for the small and the large bead with a diameter of 2 μm and 15 μm, respectively. For the largest sphere, the projection covers the slices from the bottom of the microfluidic device up to the maximum cell-depleted layer size (see dashed line in the inset image in figure 6*a*). What is interesting in these data is that around the sphere the platelet count and residence time are slightly higher than in the rest of the domain. The small sphere shows a different behaviour, because it is so small such that it is fully emerged in the CFL at the bottom of the device. There is no pattern visible in the 2D projections in figures 6*b* and 7*b* for the platelet count per second and residence time, respectively. For this smaller sphere, all values in the cell-depleted layer are taken into account as is shown in the inset images. When the shear rate on the upper plane of the geometry is increased in case of the largest sphere, the increased platelet count and residence time around the sphere become more pronounced (figure 8). At various positions (e.g. at (*x*, *y*) = (50, 8) for the 2 μm bead), a peak in platelet count is observable. These peaks are caused by platelets rolling slowly on the bottom wall. This effect is more pronounced in the 2 μm bead case, because the ROI is smaller in that case. The effect is reduced when a higher shear rate is applied to the top plane. In order to reduce this effect, the simulations need to be run for a longer period of time, however, this is computationally very expensive.

### 3.4. Shear rate and shear stresses

**Figure 9.**
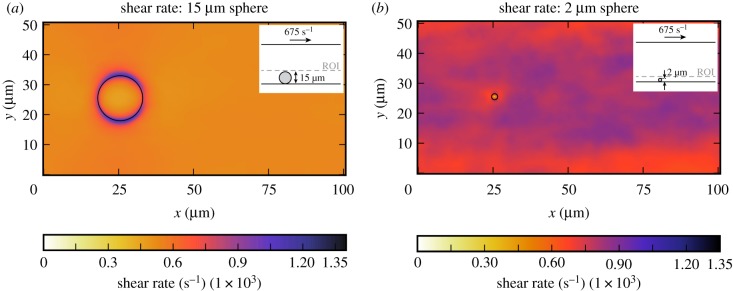
Two-dimensional projection of the averaged shear rate in the bottom layer of the flow chamber (ROI) of the bead assay simulation of the largest sphere (*a*) and smallest sphere (*b*) ($\dot{\gamma }=675\;{\text{s}}^{-1}$). The position of the sphere is marked by the black circle. The simulation details are shown in the inset image. The dashed line gives the upper limit of the layers in which the shear rate is averaged. (Online version in colour.)

**Figure 10.**
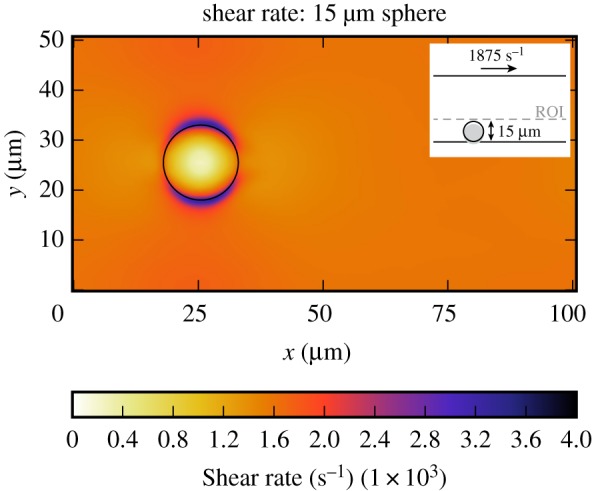
Two-dimensional projection of the averaged shear rate in the bottom layer of the flow chamber (ROI) of the bead assay simulation ($\dot{\gamma }=1875\;{\text{s}}^{-1}$). The position of the sphere is marked by the black circle. The simulation details are shown in the inset image. The dashed line gives the upper limit of the layers in which the shear rate is averaged. (Online version in colour.)

The shear rate of the fluid is measured in the bead assay experiment to study the possibility of the uncoiling of the vWF coated on the surface of the bead. The shear stress and shear rate of the fluid are further detailed in the electronic supplementary material. In figure 9, the shear rate is projected on the bottom of the microfluidic device for the case with an shear rate of 675 s^−1^ on the top plane of the domain for the smallest and largest sphere. It can be seen that the shear rates on the side of the sphere in case of the largest sphere (1200 s^−1^) are higher in comparison to the rest of the domain (600 s^−1^). In case of the smallest sphere, this difference can be neglected, since the shear rate is high everywhere in the CFL (1000 s^−1^). When a shear rate of 1875 s^−1^ is applied to the top plane a higher shear rate (4000 s^−1^) is obtained for the larger sphere (figure 10) compared to the case in which a shear rate of 675 s^−1^ was applied.

## 4. Discussion

We studied the influence of RBC on haemodynamic parameters in microfluidic devices to better understand how the transport physics influence platelet aggregation. The uniqueness of our study is that we used a cell-based blood flow framework [14] to predict the relevant haemodynamic parameters instead of a continuous blood flow model that has been used in the literature so far. One major difference that we observed is the difference in shear rate and shear stress obtained from continuous and suspension based blood flow simulations. This difference was surprisingly high and suggests that modelling blood as a continuous fluid is not suitable, directly. A continuous blood fluid model should be adopted to include a CFL and a haematocrit, to obtain the shear stresses and shear rates in microfluidic devices close to surfaces. The continuous fluid model is a good estimate of the order magnitude of shear rate in a microfluidic experiment, but exact shear rates cannot be derived from such simulations. Additionally, the shear stresses close to the wall in a continuous fluid were almost twice as high. The haematocrit of the blood was not the same everywhere, it was higher in the middle of the channel and close to zero in the vicinity of surfaces. This leads to a plug flow behaviour in the bulk of the channel, where shear stresses are low, and increased shear rates in the cell-depleted layers that act as a lubrication layer. Additionally, this migration of RBC to the middle and the presence of a CFL result in a lower local viscosity close to the channel surfaces. This explains why the shear stress is overestimated when it is modelled as a continuous fluid. This finding supports the statement of Diamond *et al*. [41] that cell-based blood flow simulations are necessary when studying the thickness of the CFL, the bulk of the blood, the drift and accumulation of platelets into the cell-depleted layer and enhanced platelet diffusivity. Most of these factors are important in platelet aggregation.

Secondly, we observed an enlarged CFL in both experiments of Tovar-Lopez *et al*. [9] and Nesbitt *et al*. [8]. In the microcontraction simulations, we obtained a CFL just after the stenosis. The enlarged CFL can be explained by the mechanism of Tovar-Lopez *et al*. [11]. They hypothesize that the wall lift effect decreases at the microcontraction and the shear-gradient lift force pushes cells to the wall. The platelets are influenced minimally by this shear gradient, as it results in negligible lift forces, thus they can maintain their trajectories. RBC are bigger and deform easier, they will be lifted from the wall and experience a change in their trajectory. The platelets can bind to vWF factor which is adhered to the PDMS surface of the microfluidic device by the Vroman effect [42], the blood plasma proteins that are most mobile will attach first to the PDMS and will be exchanged later for less mobile proteins that have more affinity with the PDMS, as explained by Tovar-Lopez *et al*. [9]. Additionally, we hypothesize that the CFL has an important role in the initialization of a platelet aggregate. In all three experiments, we observed the same behaviour of the RBC, a CFL on the location where the platelet aggregate starts to form, in combination with a large enough flux of platelets and a high enough shear rate to uncoil and activate the vWF. This immediately leads to the question as to whether such an effect could also play a role *in vivo*. Therefore, we performed a preliminary study on a 56% and a 30% vasoconstricted vessel. From this simulation, we also observed an increase in the thickness of the CFL behind the constriction. When the vessel was more constricted, the CFL increased even more (see electronic supplementary material). Additionally, we found a large enough platelet residence time and shear rate. Standard literature agrees that vasoconstriction happens upon vessel damage in haemostasis; however, they are not clear about the position in the vessel at which the vasoconstriction happens. Zucker [43] studied vasoconstriction *in vivo* in rats and found out that the vessel constricts downstream of the damaged site in the vessel. Together with our constricted vessel simulations it can be explained why vasoconstriction is happening before the damaged site and not at the site of the damage. However, this needs further investigations. Our findings are solely based and argument based on observed transport properties in our simulations. Our next step is to add platelet binding to the vessel wall and to each other to study the initial platelet aggregation in more detail, in our fully resolved cell-based blood flow model.

The increased platelet count per second and high residence time in regions with a high shear rate is our third main result. We were only interested in the platelets that are present in the CFL, because Tovar-Lopez *et al*. [11] demonstrated that platelets that contribute to the platelet aggregate are coming from the 15 to 25% of the blood stream proximal to the stenosis. When they depleted this layer from platelets, no platelet aggregate was formed. However, the plasma proteins vWF and fibrinogen were still present.

From the literature, we know that the binding time between the vWF and the glycoprotein Ib*α* is the lowest known in biology, faster than 10 μs [44]. As mentioned in the introduction, platelets can bind to vWF when the protein is uncoiled which happens above a shear rate of 5000 s^−1^ [4] for free flowing vWF and above 1000 s^−1^ [5] for bounded vWF. The shear rate in the bead assay simulations with a driving shear rate of 1875 s^−1^ is high enough for vWF factor to uncoil. Additionally, platelets are likely to be in the area of the high shear rate, since the platelet count, we obtained is high in these areas. Moreover, the residence time is larger than the time it takes to form a bond between vWF and a platelet (greater than 10 μs). The GPIb-vWF bond is reversible, which makes it possible for PLTs to roll over the vWF by forming new bonds and breaking old ones. Our hypothesis is that platelets stop rolling in the area behind the sphere due to lower shear rates and therefore they will have time to form stronger bonds with integrins.

As mentioned above, the vWF plays an important role in the formation of the platelet aggregate in high shear conditions [10]. In both experiments, we found that the shear rate is high enough to elongate the vWF that is bound to the sphere or the microcontraction wall, as explained above the shear rate should be higher than 1000 s^−1^ [5]. This elongation will take place in the CFL. It is possible that the CFL in the uncoiling of the vWF is important as it provides a high shear rate zone. When the vWF is elongated, platelets can bind to it and an platelet aggregate is formed as explained above. The formation of an enlarged CFL is supported by a stenosis or as a result of vasoconstriction, the thrombus itself, but also for example, by an atherosclerotic plaque or the struts of a stent.

Together these results provide relevant new insights into the haemodynamic environment in which a platelet aggregate starts to form. We conclude that the CFL together with an area of lower shear and an area of high shear upstream will in the presence of the vWF lead to the formation of a platelet aggregate. Nesbitt *et al*. [8] already concluded from their experiment that a local microgradient in shear rate, caused by a change in geometry or the thrombus itself, promotes platelet aggregation. However, we cannot compare our results directly to the results of Nesbitt *et al*. [8], since we are not modelling the platelet adhesion and aggregation. This will be the next step in our model development.

## 5. Conclusion

In this study, we investigated the influence of the flow dynamics on the initialization of a platelet aggregate. Based on the simulations, we conclude that an enlarged cell-depleted layer together with a high shear area in front of the CFL identifies the location where a platelet aggregate starts to form. We hypothesize that this cell-depleted layer is important for vWF to uncoil and form a platelet aggregate. Additionally, cell-based blood flow simulations are important when researchers want to assess the details of local haemodynamic parameters in their microfluidic devices, such as shear stress and shear rate.

## Supplementary Material

Movie microcontraction simulation

## Supplementary Material

Supplementary Information

## Data accessibility

This article has no additional data.
