# Supplementary material for: Identifying the start of a platelet aggregate by the shear rate and the cell-depleted layer
Source: J R Soc Interface. 2019 Oct 2;16(159):20190148. doi: 10.1098/rsif.2019.0148 (PMC6833312; doi:10.1098/rsif.2019.0148)
Supplement: Supplementary Information [file rsif20190148supp2.pdf]

# Supporting Material

B.J.M. van Rooij, G. Zavodszky, V. Azizi, and A.G. Hoekstra

Computational Science, Institute for Informatics, University of Amsterdam, The Netherlands

## General parameters of the simulations

We use a fixed lattice size of $0.5 \cdot 10^{-6}$ m in our simulations. The time step used is small since we use the immersed boundary method,. In most of our simulations we use a time step of $1 \cdot 10^{-8}$ s or $2 \cdot 10^{-8}$ s. The density and viscosity of the blood plasma, the viscosity of the blood and the parameters of the mechanical model of the red blood cell are shown in Table 1.

Table 1: The general parameters for the fluid, red blood cells and platelets of the simulations performed in this paper.

| Parameter | value |
|---|---|
| Density blood plasma [kg/$m^3$] | 1025 |
| Viscosity blood plasma [mPa·s] | 1.1 |
| Viscosity blood [mPa·s] | 2.0 |
| RBC radius [$\mu$m] | 3.91 |
| RBC volume [$\mu m^3$] | 90 |
| RBC $k_{volume}$ [-] | 20 |
| RBC $k_{bend}$ [Nm] | 80 |
| RBC $k_{area}$ [-] | 5 |
| RBC $k_{link}$ [-] | 15 |
| PLT radius [$\mu$m] | 1.25 |
| PLT volume [$\mu m^3$] | 11 |
| PLT $k_{volume}$ [-] | 100 |
| PLT $k_{bend}$ [Nm] | 250 |
| PLT $k_{area}$ [-] | 8 |
| PLT $k_{link}$ [-] | 25 |

## Shear stress and shear rate

The second-order viscous stress tensor can be calculated locally from the population independent of the velocity with the lattice Boltzmann method [1]. Additionally, there is no information required from the neighbors. The Chapman-Enskog theory can be used to determine the link between the lattice Boltzmann equation and the Navier-Stokes equations. This analysis is also needed in the derivation of the shear stress tensor in the LBM. For an incompressible fluid the shear stress tensor is given by:

$$\sigma_{\alpha\beta} = \eta(\partial_\alpha u_\beta + \partial_\beta u_\alpha) = 2\mu\mathbf{D} \tag{1}$$

with $\eta$ the dynamic viscosity. For Newtonian fluids the ratio between the shear stress and shear rate is linear and given by $\sigma_{\alpha\beta} = \dot{\gamma}\eta$. The shear stress tensor can be related to the second-order moment $\prod_{\alpha\beta}^{(1)}$ of the lattice Boltzmann populations $f_i^{(1)}$ by

$$\prod_{\alpha\beta}^{(1)} = -\frac{\tau}{3\nu}\sigma_{\alpha\beta} \tag{2}$$

with $\nu = c_s^2(\tau - \frac{1}{2})$ and $\tau$ the relaxation time. The second-order moment depends on the populations $f_i^{(1)}$ which cannot be computed. Therefore, the non-equilibrium populations $f_i^{neq}$ defined as $f_i^{neq} = f_i - f_i^{eq}$ are used instead. This is allowed according the Chapman-Enskog analysis ($f_i^{(0)} = f_i^{eq}$). The shear stress tensor in the lattice Boltzmann method is calculated by:

$$\sigma_{\alpha\beta} = -\frac{3\nu}{\tau}\prod_{\alpha\beta}^{(1)} = -\frac{3\nu}{\tau}\sum_{i}^{q-1}(f_i - f_i^{eq})c_{i\alpha}c_{i\beta} = -\frac{3\nu}{\tau}\sum_{i}^{q-1}(f_i^{neq})c_{i\alpha}c_{i\beta} = -\left(1 - \frac{1}{2\tau}\right)\sum_{i} c_{i\alpha}c_{i\beta}f_i^{neq} \tag{3}$$

In this study we will use the off-diagonal stresses from the shear stress tensor. The shear stress tensor is symmetric, therefore, we calculate the magnitude of the shear stress as follows:

$$||\sigma|| = \sqrt{\sigma_{xy}^2 + \sigma_{xz}^2 + \sigma_{yz}^2}. \tag{4}$$

The shear rate is derived from the time-averaged fluid velocity. The total shear rate is obtained by adding the shear rates in all directions.

## Cell-free layers microcontraction

The cell-free layers in the microcontraction test case with a 30 and 90 degrees stenosis and the shear rates at 1 $\mu$m from the wall at the stenosis part are shown in Fig. 1 and 2. These are prilimiary results and are not studied in full detail yet.

## Vasoconstriction

The flow in a stenosed vessel of 50 $\mu$m in diameter and a hematocrit of 27% is simulated for a constriction of 30% and 56% (see Fig. 3a). The cell-free layer is measured as a layer where the red blood cell count is 10% of the total red blood cell count. From this a contour was generated for every cross-section on which a circle was fitted to calculate the average cell-free layer. Figure 3 shows the thickness of the cell-free layer over the length of the stenosed vessel. The thickness of the cell-free layer between the apex of the stenosis and further to the end of the vessel is increasing. This observation is in agreement with the observations of the in vitro experiment of Tovar-Lopez et al. [2].

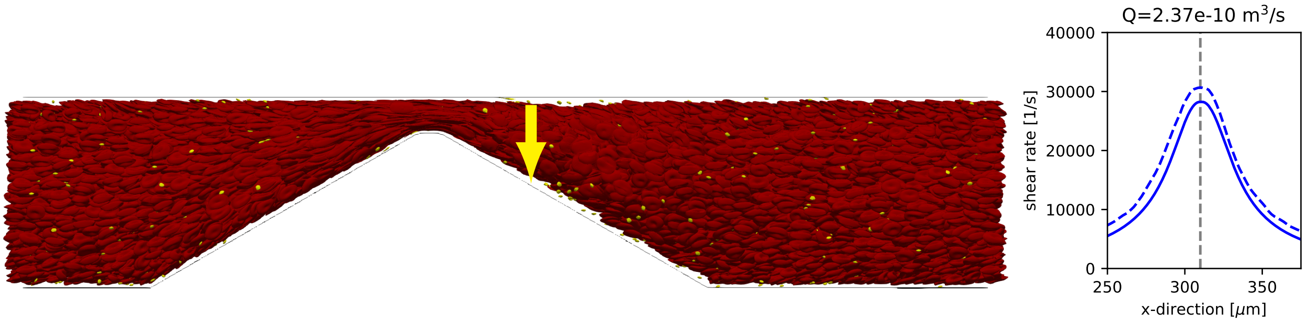


Figure 1: Left: Side view of a snapshot of the 30 degrees microcontraction simulation with blood modeled as a suspension. The red blood cells are colored red and the platelets are colored yellow. The yellow arrow indicates the position where the platelet aggregate started to form in the experiment of Tovar-Lopez et al. [2]. A cell-free layer is clearly visible on the top and bottom of the constricted flow channel. Right: Shear rate obtained 1 $\mu$m above the contraction wall in the 30 degrees geometry. The dashed line presents the simulation with blood modelled as a suspension (HemoCell) and the solid line presents the simulation with blood modelled as a continuous fluid. The vertical gray dashed line is the position of the apex of the microcontraction in the microfluidic device.

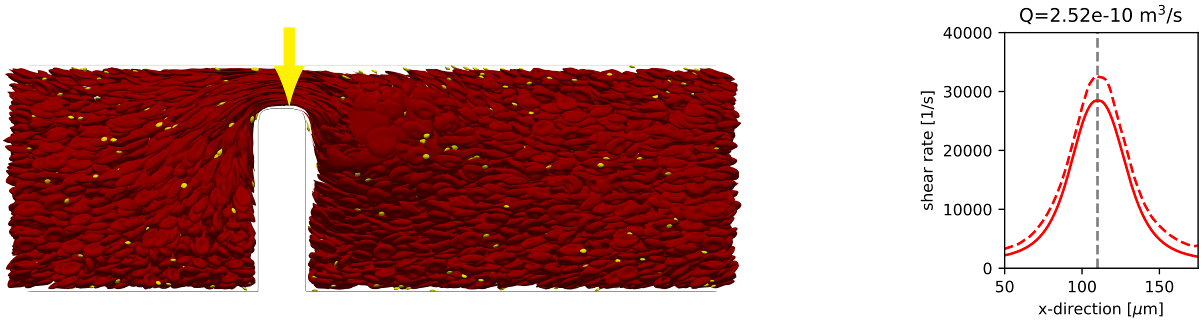


Figure 2: Left: Side view of a snapshot of the 90 degrees microcontraction simulation with blood modeled as a suspension. The red blood cells are colored red and the platelets are colored yellow. The yellow arrow indicates the position where the platelet aggregate started to form in the experiment of Tovar-Lopez et al. [2]. A cell-free layer is clearly visible on the top and bottom of the constricted flow channel. Right: Shear rate obtained 1 $\mu$m above the contraction wall in the 90 degrees geometry. The dashed line presents the simulation with blood modelled as a suspension (HemoCell) and the solid line presents the simulation with blood modelled as a continuous fluid. The vertical gray dashed line is the position of the apex of the microcontraction in the microfluidic device.

# References


[1] *The Lattice Boltzmann Method. Principles and Practice.* Springer, 2017.

[2] Francisco Javier Tovar-Lopez, Gary Rosengarten, Erik Westein, Khashayar Khoshmanesh, Shaun P Jackson, Arnan Mitchell, and Warwick S Nesbitt. A microfluidics device to monitor platelet aggregation dynamics in response to strain rate micro-gradients in flowing blood. *Lab on a Chip*, 10(3):291–302, 2010. URL `https://doi.org/10.1039/b916757a`.

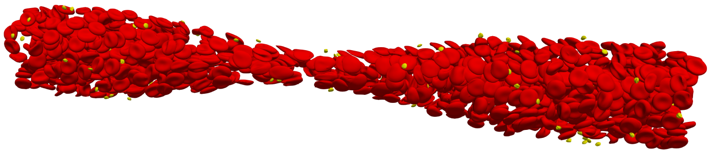

(a)

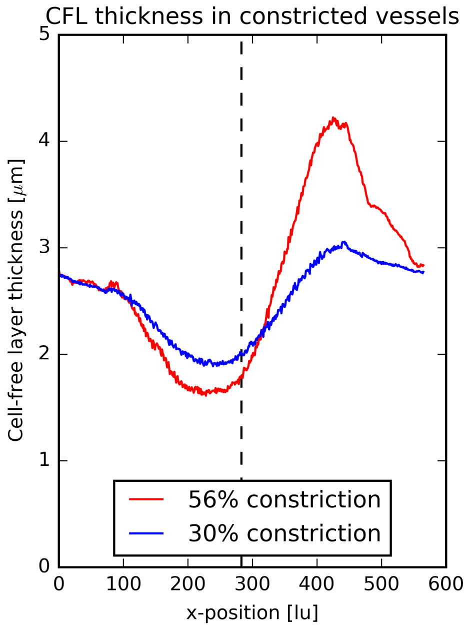


(b)

Figure 3: An visualization of the 56% constricted vessel (left) and the thickness of the cell-free layer versus the length of the vessel is shown for a vessel with 56% and 30% constriction (right). The vertical dashed line gives the location of the apex of the stenosis.
